# String Theory - The Physics of String-Bending and Other Electric Guitar Techniques

**DOI:** 10.1371/journal.pone.0102088

**Published:** 2014-07-23

**Authors:** David Robert Grimes

**Affiliations:** 1 School of Physical Sciences, Dublin City University, Dublin, Ireland; 2 University of Oxford, Oxford, United Kingdom; National Scientific and Technical Research Council (CONICET), Argentina

## Abstract

Electric guitar playing is ubiquitous in practically all modern music genres. In the hands of an experienced player, electric guitars can sound as expressive and distinct as a human voice. Unlike other more quantised instruments where pitch is a discrete function, guitarists can incorporate micro-tonality and, as a result, vibrato and sting-bending are idiosyncratic hallmarks of a player. Similarly, a wide variety of techniques unique to the electric guitar have emerged. While the mechano-acoustics of stringed instruments and vibrating strings are well studied, there has been comparatively little work dedicated to the underlying physics of unique electric guitar techniques and strings, nor the mechanical factors influencing vibrato, string-bending, fretting force and whammy-bar dynamics. In this work, models for these processes are derived and the implications for guitar and string design discussed. The string-bending model is experimentally validated using a variety of strings and vibrato dynamics are simulated. The implications of these findings on the configuration and design of guitars is also discussed.

## Introduction

The ability to explore micro-tonality and segue between pitches in a continuous manner is one element of guitar playing that sets it apart from other popular instruments where pitch is discrete. Radial pitch shifting or string-bending, the application of force to bend a string from its equilibrium position, and has become an integral part of electric guitar lead playing. Coupled with the huge array of amplification, effects and distortion options, the electric guitar can yield a vocal-like quality in lead playing, allusions to which are often made in popular culture; in Dire Strait's 1979 debut single “Sultans of swing”, songwriter Mark Knopfler refers to a jazz guitarist as being “strictly rhythm, he doesn't want to make it cry or sing”. Eric Clapton's thick guitar tone and use of vibrato is referred to by guitarists as the “woman tone”, which he famously contributed to the Beatles's classic “While my guitar gently weeps”. These are but some examples - An accomplished guitarist's tone and vibrato can be so intrinsic to that player that their idiosyncratic sound is as distinctive as a vocalist's to a trained ear.

From a physical perspective, the basic acoustics of string instruments have been well studied for centuries. There has been considerable analysis of the physics of violins, practically all of which is equally applicable to guitar. Much of this work is concerns the mechano-acoustic properties of vibrating plates and applications of Chladni's Law [1]. Considerable investigation has been undertaken on the mechanics of acoustic guitar construction [2] and resonances [3], [4], as well as analysis into the tonal effects of removable back-plates [5]. Similarly, other research [6] has examined the acoustics of classical guitars and the effect of bridge design on top plate vibrations. Such analysis is understandably critical for acoustic instruments, but perhaps less for electric guitars which rely on suitable valve or solid-state amplifiers to project their sound. As a consequence, some research pertaining explicitly to the electric guitar has concentrated on the mechanical-electrical properties of electric guitar pick-ups [7]. Other research has examined the challenge of digitally simulating the tonal quality of electric guitars in syntheised instruments [8]–[10]. As guitars provide a tangible example of the many important physical principles, some literature on the subject is dedicated to the pedagogical advantages of using guitars as demonstrations of crucial acoustic principles such as standing waves [11].

Yet despite the significant volume of research dedicated to guitar acoustics, there appears to be a paucity of research concerning the physics of electric guitar playing and the underlying physical principles of the distinct techniques which influence the instrument's unmistakeable tonality. As the electric guitar has become ubiquetous in rock, pop, metal, jazz and blues, it has developed a range of techniques which further distinguish it from its musical forebears. String-bending and vibrato add much to a guitarist's palette, and as these techniques are heavily influenced by the physical constraints of the guitar and strings used, the underlying mechanics are worthy of analysis. Guitarists also use a wide variety of *legato* techniques to articulate their playing; these include hammer-ons, where a fretted string is picked and another one sounded by coming down sharply on it with the fretting hand, resulting in a smoother sound that would result from merely picking both notes. The opposite technique is a pull-off, where a picked note is released and a lower one sounded. A fusion of both these techniques practically unique to the guitar is tapping, where both fretting and picking hands are used to ‘articulate’ a flurry of notes. This is a staple of modern lead guitar playing, popularised by guitar virtuoso Edward Van Halen in the late 1970s. For these techniques, the fretting force required to ‘sound’ a note with or without picking becomes an important limiting factor and influences a player's choice of string and guitar set-up. Unlike more traditional instruments, there is also wide scope for modification and extension of tonal range by using external hardware. One example of this is the vibrato system or “whammy-bar” which many guitars opt for; these are mechanical systems for adding extra vibrato and come in a variety of designs. Rather misleadingly, they are sometimes referred to as tremolo bars, which is an unfortunate misnomer as tremolo is modulation of volume rather than pitch. Such units dramatically increase the sonic scope of the instrument and have been employed by famous players such as Dave Gilmour, Brian May, Steve Vai, John Petrucci, Frank Zappa and Joe Satriani amongst others.

As the electric guitar has such a wide range of techniques and modifications, it is worthwhile to analyse these factors from first principles, and examine what implications these elements have on guitar design and playing. This work will concentrate on some of the unique principles of electric guitar playing, such as string-bending, vibrato, fretting force and whammy-bar effects. String design is discussed, and string-bending factors experimentally examined. Vibrato and related effects are simulated and discussed.

## Materials and Methods

### 0.1 String bending

**Figure 1:**
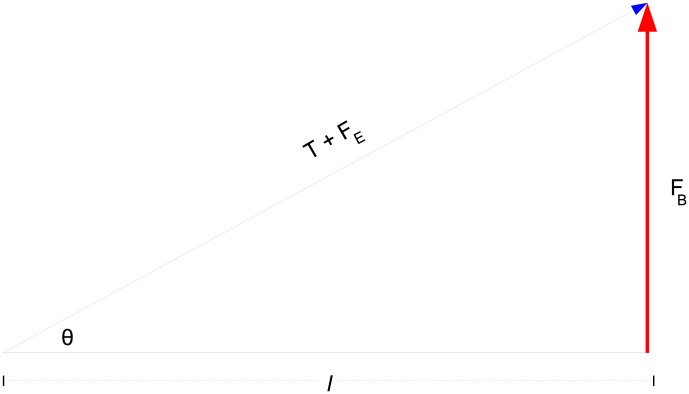
Force diagram for a bent string.

For a stretched string, the fundamental frequency may be derived from the one dimensional wave equation, and is given by 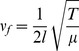
(1)where 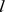 is the length of the vibrating element, 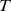 is the string tension and 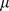 the linear density or mass per unit length of the string. The technique of string-bending is the application of a another force perpendicular to the fretboard - an illustration of this in shown in figure 1 where a bending force, 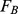, is applied to a string. This applied force causes a slight increase in the vibrating length, which would act to lower the pitch if not for the net increase in string tension due to the extension force acting along it, 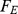. This acts to raise the pitch. Assuming the distance from the string to the fretboard is small so that length increases in this plane are negligible, the fundamental frequency of a bent string in terms of the extension force 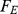 and the bend angle 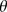 is then 
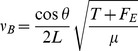
(2)


The bend angle is not independent of the extending force, and the relationship between the twain can be explicitly calculated; the string is elongated along its vibrating length when bent, and by Hooke's law the force and extension are related by 
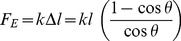
(3)


Hooke's stiffness can be related to the Young's modulus (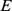) and cross-sectional area of the string 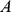, and (3) can be rewritten in these terms as 
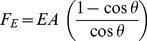
(4)


Thus, the frequency of a bent string is described by first approximation to 
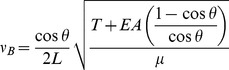
(5)


Strictly speaking, (5) holds when 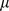 is constant. This is defined as mass (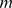) per unit length; Hooke's law states that the string stretches in proportion to the applied force. Defining 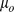 as the mass per unit length with no force applied, then the complete expression the bent frequency is 
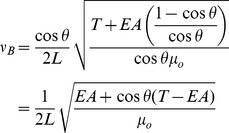
(6)


In practice, bend angles that can be employed by most players with a standard configuration are unlikely to exceed even 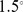. Guitar strings tend to be composed of steel or steel alloys and as such can have a Young's Modulus of up to 200 GPa [12], and so equation 6 predicts that even these relatively small bend angles can have a substantial effect on pitch. It is important to note that guitarists can bend either “up” or “down”, yet the net effect is always to raise pitch relative to an unbent string and it is the magnitude rather than the direction of the bend that is relevant. The reason for this is explored in the discussion section. Examples of string-bending are included in the supplementary material for reference (Video S1).

### 0.2 Fretting-Force and Bending force

**Figure 2:**
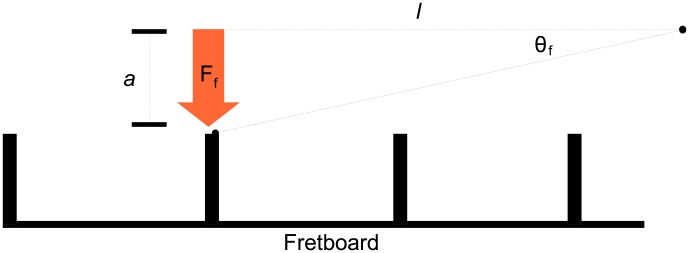
Fretting force diagram.

On a fretted guitar, the string is depressed until it makes contact with the fret marker. The distance between the fingerboard and string is called the “action” of a guitar. Generally, low action is considered preferable as lower action guitars are easier to fret, but if the action is too low the string may buzz due to collisions with fret-markers or the fretboard. The force required to fret is not only important for physically sounding notes, but is also vital for some prominent guitar techniques such as hammer-ons and tapping, examples of which are included in the supplementary material (Video S2). If we denote the action as the distance from the string to the fret-marker 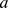 and the angle the depressed string makes with the bridge as 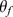 as depicted in figure 2, then the effective tension on the string can be resolved to yield an expression for fretting force of 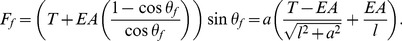
(7)


Similarly, the force required to bend a string to the angle 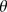 as in equation 6 is given by 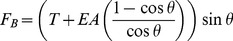
(8)


### 0.3 Vibrato

Vibrato is a regular pulsating change of pitch, and adds expression to a note. It is remarkably important in guitar music, and can be so utterly unique to different players it can be used in some cases to distinguish them by ear. In electric guitar playing in pop, rock and metal, vibrato is often produced by the player modulating the angle of the string, essentially bending it up and down to vary the pitch. This can be modelled by modifying equation 6 with the bend angle as a function of time, denoted 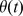. In this case, the rate of vibrato is given by the first derivative of frequency with respect to time by 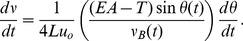
(9)


In classical guitar playing, most vibrato is produced by “axial” vibrato techniques, where the tension is altered on a fretted note by modulating the tension applied so that the bend angle is kept at zero. In this case the variable is tension and the process can be modelled by modifying equation 1 with a time-varying tension for 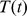. The vibrato rate is then given by
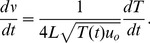
(10)


In practice, guitarists will have vastly differing approaches to vibrato - it is not uncommon to hear guitarists describe to a particular vibrato as “wide” or “narrow”, in reference to how much angular or tensional displacement the player is applying. Similarly, other vibratos may be described as “fast” or “slow”, depending on the rate at which the player modulates the vibrato. Like bending, vibrato will not lower the pitch from the resting pitch. Examples of both vibrato types are included in the supplementary material (Video S3).

### 0.4 Whammy-bar dynamics

Whammy-bars come in a variety of designs, and famous models include the Bigsy Vibrato Tailpiece, the Fender Strat Tremolo and the Floyd Rose locking tremolo. These work by changing the string tension with a controlling lever at the bridge. In this case, the vibrato rate can be described very simply by modifying equation 1 to yield 
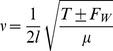
(11)where 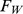 is the force applied to the vibrato arm. It is important to note that on many models tension can be either decreased or increased, with an increase in tension acting to increase pitch and vice versa, encapsulated in the 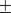 term in equation 11 for an increase and decrease in pitch respectively.

### 0.5 Experimental method for investigation of string-bending

**Figure 3:**
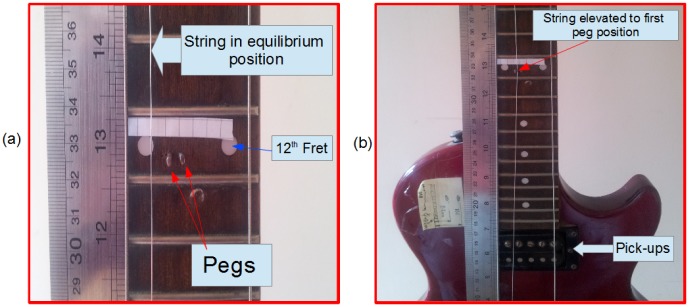
Modified experimental guitar with two bending pegs inserted at 12th fret (a) String in equilibrium position, string unbent. (b) String affixed above first fretting peg, a vertical displacement of approximately 7.5 mm from resting position.

**Figure 4:**
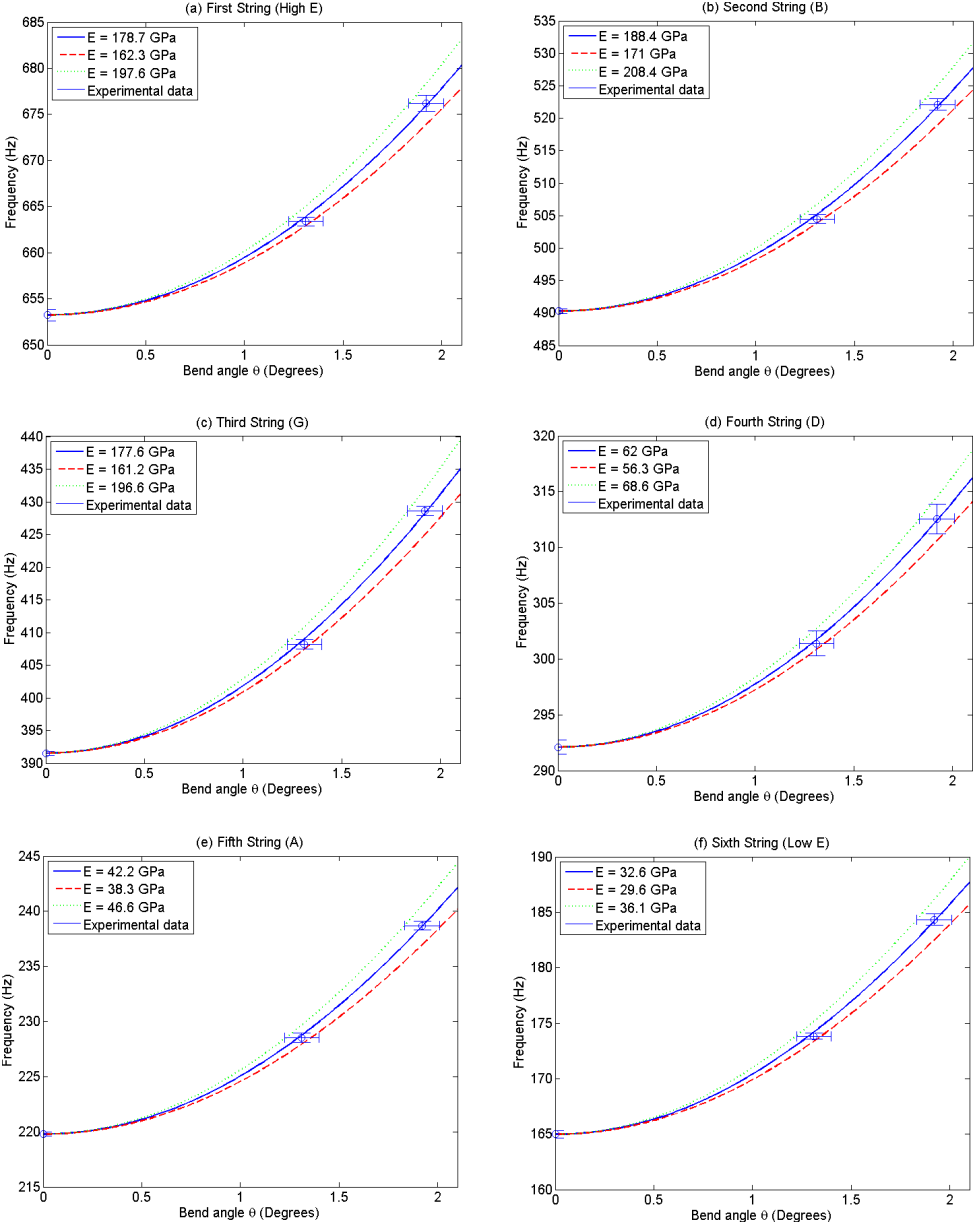
Results for test string on experimental guitar rig. The highest three strings depicted in (a), (b) and (c) had approximately the same Young's modulus, close to that of steel. For composite wound strings depicted in (d), (e) and (f), this was markedly lower and decreasing. Best fits are shown for all strings with the solid line, the the dotted and dashed lines depict best fits if bend displacement is plus or minus 0.5 mm from measured value.

If the expression for the frequency of a bent string given by 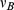 as derived in (6) describes string bending well, then the expression for fretting force 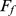 and rate of change of pitch 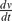 follow from this. To investigate this experimentally, a fixed bridge Gibson Epiphone special was stripped down (Gibson, Nashville, TN) and modified by the addition of of two holding pins at the 12th fret position, approximately 328 mm from the bridge of the instrument. The guitar output was connected to a chromatic tuner and then to an undistorted amplifier. A microphone was placed at the amplifier speaker and output frequency was measured using the *Waves* software package (*Cohortor.org*). On the modified guitar, taut strings in the 1st and 6th positions were used as supporting wires to prevent the nut from slipping when the test string was replaced. The test string was loaded in the 5th string position for each test, as depicted in figure 3. The holding pins were placed at 7.5 mm and 11.1 mm respectively above the equilibrium position of the test string as depicted in figure 4, yielding bend angles of 0°, 1.31° and 1.94° approximately. These positions were chosen to give a range of realistic bend angles below the threshold which strings may snap.

Ernie Ball Regular Slinky gauge strings (Ernie Ball, California) were then individually loaded in the test rig and tuned to their respective concert pitchs using a chromatic tuner. The pitch of the individual test strings were recorded unfretted, fretted at the 12th fret position and then respectively fretted on the first and second holding pin for each string in the set. The linear density of the strings was determined by cutting the strings into 30cm sections and then weighing them on a calibrated weighing scales. String diameter and area was measured using a digital micrometer screw gauge. The Slinky regular gauge strings were tempered for standard concert pitch for a six string guitar with gauges, with gauges of 46, 36, 26, 17, 13 and 10, corresponding to low E string, A string, D string, G string, B string and high E string respectively. Of these, the 17, 13 and 10 gauge strings were plain high carbon steel wire plated with tin. The 46, 36 and 26 gauge strings had a high carbon steel core and were wound with nickel. Measured properties are shown in table 1. When loaded in the test rig, the tension estimate for each string was obtained by manipulating equation 1. The action distance from test string to fret marker 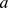 was 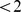 mm for all string positions along fretboard, yielding small values of 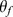 so effects in this plane were expected to be negligible.

**Table 1:** Measured string properties.

String gauge	Concert Pitch	Area( 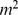 )	Linear density 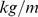
10	High E	5.1338 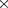 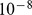	3.94 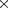 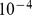
13	B	9.0259 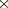 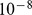	6.94 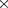 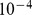
17	G	1.4816 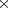 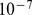	1.14 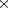 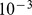
26	D	3.5503 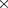 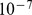	2.33 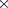 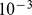
36	A	6.788 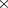 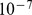	4.32 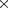 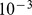
46	Low E	1.1004 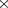 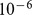	6.97 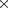 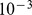

## Results

### 0.6 String-Bending analysis

Results of the string-bending analysis for all string types are shown in figure 4 with best fit values of 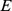. The mean and standard deviation of frequency measured over multiple runs is plotted in vertical axis. In the horizontal axis, the angular values measured were plotted as the mean with a potential measurement uncertainty of 0.5 mm each side and the corresponding angular displacement used to establish upper and lower bounds on the experimental estimates of 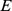. For the highest pitched strings composed of a single steel wire with tin plating, the estimated Young's modulus and range estimates were approximately constant at around 177.6–188.4 GPa, close to the expected limit for steel [12]. For the lower pitched composite strings, the estimated elastic modulus was considerably lower and decreased with pitch. Goodness of fit in each case was high, indicating strong agreement. The experimental results obtained are depicted in table 2

**Table 2:** Experimental results for string types.

String gauge	Estimated 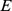	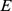 range ( 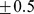 mm)	Estimated 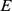 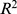
10	178.7 GPa	162.3–197.6 GPa	0.9987
13	188.4 GPa	171–208.4 GPa	0.9988
17	177.6 GPa	161.2–196.6 GPa	0.9988
26	62 GPa	56.3–68.6 GPa	0.9994
36	42.2 GPa	38.3–46.6 GPa	0.9997
46	32.6 GPa	29.6–36.1 GPa	0.9993

### 0.7 Vibrato simulation

**Figure 5:**
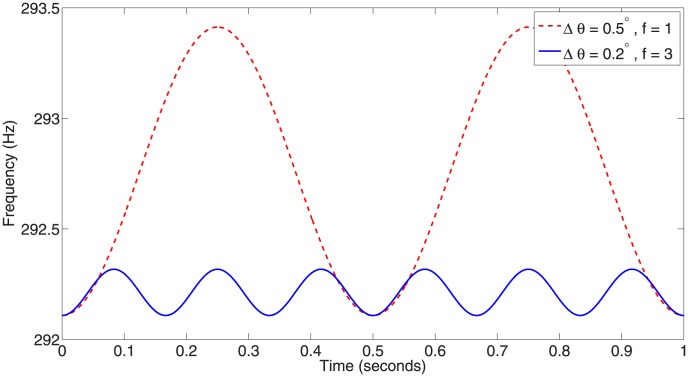
Simulation results for a fast, narrow vibrato and a slow, wide vibrato.

In practice, vibrato will vary massively with player technique but despite this it is possible to simulate the general conditions under which vibrato occurs. A simple model of vibrato due to radial displacement was simulated by using a time-dependent sinusoidal function given by 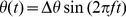
(12)where 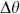 is the maximum displacement angle and 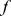 is the vibrato frequency. For this simple simulation, a narrow, fast vibrato and a wide, slow vibrato were simulated by selection of appropriate parameters. Illustrations of this are shown in figure 5. Despite the relative simplicity of the function in (12), resultant vibratos were aurally similar to real guitar vibratos, and examples are included in the supplementary data (Video S4).

## Discussion and Analysis

Results from experimental data for string-bending over the range of gauge types in this study strongly suggest that the string bending equation in equation 6 describes the physio-acoustic properties of bending very well. Despite the small angles typically involved in string-bending, these small perturbations from equilibrium position have a distinct perceptible effect on pitch, illustrating the micro-tonality the guitar is famous for. As can be seen in results figure 4, string-bending is not linear; while very small angles may only add subtle variations in pitch, pitch increases markedly and non-linearly beyond these small angles. This may be part of the reason why string-bending can be a technique novice guitarists find difficult to master, as a certain amount of intuition is required, especially in techniques such as pre-bends, where notes are bent before being picked and allowed relax to equilibrium.

While string bending tends to act to increase pitch, one could in theory envision a situation where bending a string might act to lower pitch so that 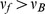. However, the derived equations explains why this doesn't happen in practice: if it did occur, then it would imply 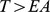 in such a domain. For typical guitar string material, this would require a tension force far beyond the string limit or an impractically miniscule cross-sectional area. In reality, guitar strings are subject to the constraint 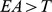, thus bending always acts to increase pitch.

**Figure 6:**
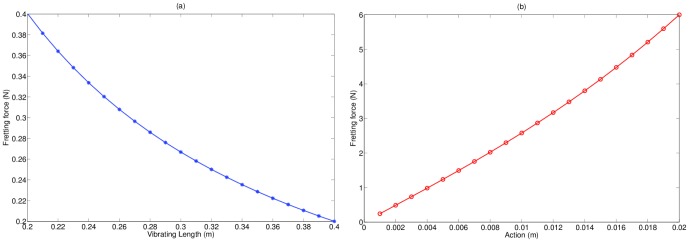
Variation of fretting force with (a) vibrating string length (b) fret-board action. Shortening the vibrating string length increases the fretting force required, so higher frets are more difficult to push down than lower ones. Fret-board action has the greatest influence on fretting force, and even relatively small differences in displacement from the fret-marker can have substantial effect on effort required. In these examples, 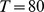 N and 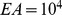 N.

Fretting force is also of considerable interest, as it heavily influences both guitar design and player style. In the form derived in this work, it can be considered a function of vibrating string length and fret-marker action. The relative effects of these parameters are shown in figure 6. In general, string fret-board action has the greatest influence on fretting force required to sound a notes, having a much more pronounced effect than string length. Shorter vibrating string lengths require greater force to fret, rendering higher frets more difficult to finger. While string-bending pitch is angle-dependent, the angles involved are small so that 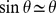. This results in string bending force being approximately linear with angle in the domain of interest so that a bend angle of 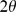 requires approximately double the bending force required to bend the string an angle of 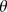. As discussed previously, while the force may be approximately linear the resultant frequency is not and in general 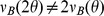.

The human ear is remarkably good at detecting different pitches, and in clinical studies the frequency resolution of the human ear is about 3.6 Hz [13]. Even smaller pitch changes can be detected as beat frequencies, a perceived periodic change in volume caused by the interference of two tones of slightly different frequency [14]. In this analysis, the action between the fret-board was small relative to the string length so that effects in this plane could be ignored. This is usually the case in practice, but it is possible to use the theory in this study to investigate the values of 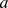 at which this would have a distinct effect on pitch and could no longer be ignored. Denoting the resolvable frequency change as 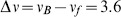 Hz, then we can infer what values of 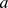 would begin to have a perceptible effect on pitch. For the strings in this study, this value is typically 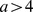 mm, which would correspond to a high action guitar. Were this the case, then not only would this set-up potentially affect the intonation of the instrument, it would render it rather difficult to play. It is worth noting that on a guitar neck the strings are rarely absolutely parallel to the fingerboard, and are usually slightly higher towards the bridge. In these situations, action will increase from the nut (neck of the guitar) to the bridge.

Two distinct methods of producing vibrato are discussed in this work, and a method of simulation outlined and implemented. Even with the simplistic approximation given in equation 12, the resultant variation in pitch is a good approximation of a real vibrato. In reality, a player's vibrato will likely be of a much more complicated form, but the underlying physical principles should remain the same.

While modelling the effects of string manipulation on pitch is in itself an interesting exercise in applied physics, this type of analysis may have much application in digital instrument modelling. As is clear in this work, mechanical changes have a distinct effect on pitch and it is this relationship that gives the guitar such an expressive palette. To better model digital instruments, the interactions discussed in this work could be taken into account and implemented with relative ease. The other application of this work may be in guitar and string design. Intrinsic physical properties of the string material, such as its Young's modulus, have a profound effect on vibrato, bending and fretting force. Similarly, design properties such as cross-sectional area and linear density also have an effect, and explicitly detailing the relationships between these factors may facilitate in improving string design. This study also has implications for guitar configuration, particularly the effects of fret-board action on play-ability and resultant pitch. The experimental procedure in this work could be readily replicated with equipment available in most university physics labs, and as the derived models involve applied physics principles, acoustics and mechanical properties of string materials, this could serve as a useful pedagogical investigation of these phenomena for science students.

The current work does not consider the interplay of the pick and striking dynamics on the string, though much of the current analysis on strings would be applicable to such situations and may inform work on this factor.

## Conclusions

The models in this work describe the behaviour of guitar strings under mechanical manipulation, and the effects of such manipulation on pitch and resultant tone. String-bending, vibrato and fretting force are all considered. The effects of string-bending are experimentally validated for a range of different gauges, and found to agree well with the model proposed. The implications of these factors on guitar construction and configuration are discussed in some depth, as is the potential application of such analysis to digital simulation of guitars. The implications of this analysis for guitar configuration and string design is also discussed.

## Supporting Information

Video S1 **A video demonstration of string bending.**
(MP4)

Video S2 **A video demonstration of legato techniques.**
(MP4)

Video S3 **A video demonstration of vibrato techniques.**
(MP4)

Video S4 **Midi files of (a) Pure tone at 292.108 Hz (b) Simulated axial “bending” vibrato at 292.108 Hz with 3 Hz bending frequency at an angle of 0.2 degrees.**
(WMV)
